# Potentiating anti-inflammatory and antioxidant effects in vitro: the combined action of zofenoprilat and nebivolol

**DOI:** 10.1007/s43440-026-00833-x

**Published:** 2026-02-03

**Authors:** Eleonora Maceroni, Annamaria Cimini, Michele d’Angelo, Marta Sofia Scenna, Suada Meto, Simone Baldini, Paolo Fabrizzi, Giovambattista Desideri, Vanessa Castelli

**Affiliations:** 1 Department of Life, Health and Environmental Sciences, University of L’Aquila, 67100 L’Aquila, Italy; 2 Menarini Group, Via Sette Santi 1–3, 50131 Florence, Italy; 3 Department of Clinical, Internal Medicine, Anesthesiologic and Cardiovascular Sciences, Sapienza University of Rome, 00161 Rome, Italy

**Keywords:** Hypertension, Vascular health, Oxidative stress, Inflammation, Cardiovascular diseases

## Abstract

**Background:**

The present study investigated the combined effects of zofenoprilat (ZOFE) and nebivolol (NEBI) on endothelial function, focusing on their anti-inflammatory and antioxidant properties. The purpose was to evaluate whether these drugs, commonly used in clinical practice, offer a synergistic therapeutic strategy for managing hypertension and protecting vascular health. ZOFE, an ACE inhibitor, demonstrated significant anti-inflammatory activities by reducing inflammatory cytokines, thereby mitigating vascular inflammation, a key factor in hypertension and atherosclerosis. NEBI, a third-generation beta-blocker, exhibited strong antioxidant effects by enhancing nitric oxide (NO) levels, crucial for maintaining endothelial function and reducing oxidative stress.

**Methods:**

The potential effect of ZOFE and NEBI treatment was evaluated using human umbilical vein endothelial cells (HUVEC) as a model. Specifically, cells were challenged with tumor necrosis factor-α (TNF-α) to induce endothelial dysfunction. Subsequently, cell viability, NO production, protein levels of superoxide dismutase (SOD) and catalase (CAT), enzymatic activity of SOD and CAT, intracellular levels of glutathione (GSH), inflammatory status, and levels of interleukin-6 (IL-6) monocyte chemoattractant protein-1 (MCP-1), macrophage inhibitory cytokine-1 (MIC-1), and the active form of nuclear factor kappa B (p-NFκB), were analyzed.

**Results:**

Our results showed that NEBI significantly counteracted oxidative stress, increasing the main antioxidant defenses (SOD, CAT, and GSH). The combination of ZOFE and NEBI resulted in a potentiated effect, enhancing both anti-inflammatory and antioxidant activities. This dual mechanism of action provides a comprehensive approach to protecting endothelial cells and improving vascular function. The combined therapy not only lowered blood pressure more effectively but also offered greater protection against endothelial damage compared to monotherapy with either drug alone. These findings suggest that the combination of ZOFE and NEBI could be particularly beneficial for patients with hypertension, especially those with coexisting inflammatory and oxidative stress-related conditions.

**Conclusions:**

This combination therapy, by addressing multiple pathogenic pathways simultaneously, could potentially be beneficial in patients with cardiovascular risk conditions. In conclusion, the combination of ZOFE and NEBI offers a potentially promising therapeutic approach for managing hypertension and protecting vascular health, aiming at improving clinical outcomes for patients with cardiovascular diseases.

**Clinical trial number:**

Not applicable.

**Supplementary Information:**

The online version contains supplementary material available at 10.1007/s43440-026-00833-x.

## Graphical Abstract

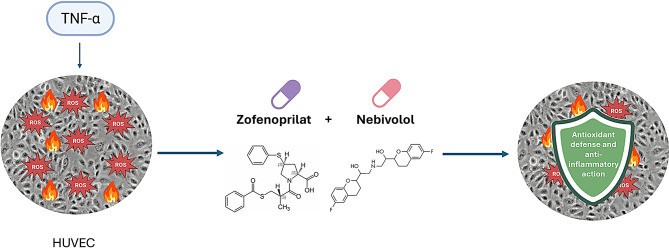

## Introduction

Hypertension is a chronic medical condition characterized by persistent elevation of the blood pressure in the arteries. In the absence of intervention, this condition can result in severe health complications, including heart problems, stroke, and kidney dysfunction, if not properly managed. Hypertension is often referred to as the “silent killer” because it may not manifest evident symptoms until substantial damage has already occurred. The multifaceted clinical status of patients with hypertension may be attributable to reactive oxygen species (ROS)-mediated oxidative stress. Nitric oxide (NO) is a paracrine factor produced by endothelial cells that has been shown to relieve ROS-mediated oxidative injury [1].

In addition, increasing studies indicated that hypertension is associated with a chronic inflammatory state, characterized by the transmigration, accumulation, and initiation of inflammatory cells, and the pro-inflammatory cytokines and free radicals released by activated innate immune cells and endothelial cells [2, 3]. Although inflammation and oxidative stress are linked to hypertension, the underlying mechanisms are not completely understood.

Nebivolol (NEBI) is a third-generation beta-blocker that exhibits high selectivity for β1-adrenergic receptors. It is currently approved for the treatment of essential hypertension and of stable mild and moderate chronic heart failure, in conjunction with standard therapies in elderly patients ≥ 70 years. The distinct pharmacologic profile of nebivolol is related to a multitude of hemodynamic consequences: (1) β1-blockade, which declines resting and exercise heart rate, myocardial contractility, and both systolic and diastolic blood pressure; (2) NO-mediated vasodilation that leads to peripheral vascular resistance, an increase in stroke volume and ejection fraction, and maintenance of cardiac output [4]; (3) vasodilation and reduced oxidative stress that are thought to contribute to the neutral and possibly beneficial effects of nebivolol on glucose and lipid metabolism [5, 6]; and (4) decreased platelet volume and aggregation [7, 8].

Owing to these characteristics, NEBI not only reduces blood pressure but also provides additional cardiovascular advantages [9]. The vasodilating properties of nebivolol, along with its peculiar profile, make NEBI a favored option when a beta-blocker is selected according to current European Guidelines for the management of hypertension [10, 11].

Zofenoprilat (ZOFE) is an active metabolite of zofenopril, an angiotensin-converting enzyme (ACE) inhibitor. The mechanism of action of this pharmaceutical agent involves the inhibition of the enzyme responsible for the conversion of angiotensin I to angiotensin II, a potent vasoconstrictor. By reducing the levels of angiotensin II, ZOFE helps to relax blood vessels, thus decreasing blood pressure and the workload on the heart [12].

Both NEBI and ZOFE are effective in managing hypertension, but they work through different mechanisms. NEBI primarily affects the heart and blood vessels through beta-blockade and NO release, while ZOFE targets the renin-angiotensin system to prevent vasoconstriction and facilitate vasodilation.

Notably, it has been reported that ZOFE was more efficient than another sulfhydryl-containing ACE inhibitor, captopril, and two non-sulfhydryl-containing ACE inhibitors (enalaprilat and lisinopril) in increasing NO metabolite production [13], which is essential for overall health, particularly cardiovascular homeostasis.

Both NEBI and ZOFE belong to major classes of anti-hypertensive drugs, which can be, in principle, combined together - as per ESH 2023 and ESC 2024 clinical guidelines currently in force [10, 11], and there is evidence of their consolidated use in extemporaneous combination in common clinical practice [14].

Although NEBI and ZOFE are well established in the clinical management of hypertension, data regarding their combined effect at the cellular level remain limited. Specifically, this study aims to fill the knowledge gap related to the synergistic impact of these drugs on endothelial dysfunction mediated by oxidative stress and inflammation.

Human umbilical vein endothelial cells (HUVECs) were selected as the experimental model. These primary cells, isolated from the endothelium of umbilical cord veins, are widely used to investigate endothelial cell function and pathology. HUVECs play a crucial role in maintaining vascular homeostasis and are essential for understanding mechanisms underlying cardiovascular health and disease.

Inflammatory mediators, such as tumor necrosis factor-α (TNF-α), have been shown to trigger the activation of endothelial cells, eventually resulting in cellular impairment that may contribute to cardiovascular and metabolic vascular disorders.

When challenged with TNF-α, HUVECs exhibit inflammatory and oxidative responses that closely mimic vascular alterations observed in hypertensive conditions.

Thus, in this study, HUVECs were challenged with TNFα to induce endothelial dysfunction and then treated with NEBI and ZOFE.

Specifically, we investigated the underlying cellular and molecular mechanisms of NEBI and ZOFE and whether their combination can have a synergistic effect in managing hypertension due to their complementary mechanisms of action. In the first set of experiments, we evaluated the mechanisms underlying endothelium-dependent NO liberation by NEBI and ZOFE alone and in combination by examining NO production in HUVECs. Subsequently, we investigated whether the NEBI and ZOFE combination had protective effects against oxidative damage and inflammation induced by endothelial dysfunction.

## Materials and methods

### Cell culture

Primary human umbilical vein endothelial cells (HUVECs # C2519A Lonza, US) were cultured in the recommended complete endothelial growth medium supplemented with ECG (#CC-3162 Lonza) at 37 °C in a 95% air 5% CO_2_ humidified incubator. Cells between passage 5 and 10 were employed in all experiments, and the seeding density for all experiments performed was 10’000 cells/cm^2^.

HUVECs were incubated either with ZOFE (zofenoprilat is the active form of zofenopril, purchased from Santa Cruz, CAS 1329569-13-2), or NEBI (Menarini Industrie Farmaceutiche Riunite S.r.l.) diluted in culture medium(#CC-3162, Lonza), or the combination for various times up to 24 h. All compounds were used at 10 − 8 M as previously reported by [13]. Based on the nitric oxide (NO) results, 24 h were selected for the following experiments. Tumor necrosis factor-α (TNFα, #SRP3177 Sigma-Aldrich, US), a mediator of endothelial dysfunction, at 10ng/ml was used alone (untreated) and in combination with the tested drugs for 24 h.

### Viability assay

The dose-response curve of HUVECs upon NEBI or ZOFE alone and in combination for 24 h was performed. Specifically, cell titer one solution cell proliferation assay (#G3581 Promega, USA) was employed directly in a cell culture 96-well plate, and the formazan production was measured at 492 nm as previously described [15] using a Spark microplate reader (Tecan, US).

### Nitric oxide assay kit

NO production by HUVECs was determined by assessing the concentration of NO metabolite in fresh samples using Abcam assay (#ab65328) supplemented by a 10 kDa spin column (#ab93349, Abcam). Briefly, the samples and standards were added to wells, followed by nitrate reductase and enzyme cofactor addition, and then incubated for 1 h at room temperature to convert nitrate to nitrite. Finally, the enhancer provided in the kit was added, and the microplate was read at 540 nm using a Spark microplate reader. Data are reported as % of control.

### Oxidative stress defense

Oxidative stress defense (Catalase, SOD1) western blot cocktail was used to analyze oxidative stress from cell lysate (#ab179843, Abcam).

Cells were gently washed with cold 1× phosphate-buffered saline buffer and scraped with cold RIPA buffer (#89901 Thermo Scientific, US) with freshly added protease and phosphatase inhibitors (#1861284 Thermo Scientific, US) and incubated on ice for 1 h. Lysates were centrifuged at 14,000× g RPM for 30 min at 4 °C. Protein concentration was assayed by the BCA kit (#23227 Thermo Scientific, US).

Then, an equal amount of proteins, 30 µg/lane, was resolved in bolt gradient precast gels (#NW04122BOX Thermo Scientific, US) and run following the manufacturer’s protocol. Proteins were transferred to polyvinylidene difluoride membranes and then blocked in blocking buffer (#37543 Thermo Scientific, US). Membranes were then incubated with the antibody cocktail and housekeeping protein and visualized with SuperSignal™ West Pico PLUS (#34580, Thermo Scientific) following the manufacturer’s protocol. Different expositions were acquired using the iBright device (Thermo Scientific).

### Superoxide dismutase (SOD) enzyme activity

The total SOD activity was measured by an enzymatic method using an SOD assay kit (#ab65354) purchased from Abcam, UK. SOD activity was assessed by measuring the rate of reduction of WST-1 (2-[4-iodophenyl]-3-[4-nitrophenyl]-5-[2,4-dis-ulfophenyl]-2 H-tetrazolium, monosodium salt), which produces a water-soluble formazan dye when reduced by a superoxide anion. The absorbance of WST-1 was measured at 450 nm using a Spark microplate reader. The total SOD activity measured was determined by measuring the decrease in color development at 450 nm compared to the control.

### Catalase (CAT) enzyme activity

CAT enzyme activity was evaluated (#ab83464, Abcam UK). Specifically, the catalase contained within the sample reacts with hydrogen peroxide, resulting in the production of water and oxygen. The unconverted hydrogen peroxide reacts with a probe to generate a product that can be measured colorimetrically at OD 570 nm. In summary, samples and standards were prepared and subsequently loaded into wells. Hydrogen peroxide was then added to the wells, and the mixture was left to incubate at 25 °C for 30 min. Finally, the stop solution was added, followed by the developer mix, and incubated for 10 min at 25 °C. The plate was measured at a wavelength of 570 nm using a spark microplate reader.

### Intracellular glutathione (GSH) detection assay kit

Intracellular glutathione detection assay kit (#EIAGSHC, Thermo Scientific) was used for evaluating intracellular glutathione (GSH) concentration. This assay employs a colorimetric substrate that reacts with the free thiol group on GSH to yield a highly colored product. The measurement of the concentration of GSH can be conducted by using a spark microplate reader at 405 nm. Data are reported as µM interpolated with the standard curve.

### Inflammatory status

Proteome profiler human XL cytokine array kit (#ARY022B, R&D, USA) was used. Conditioned media were collected from TNFα-challenged cells (untreated) and treated cells and loaded onto spotted membranes, with the volume of sample adjusted as recommended by the manufacturer (500 µL). The reagents were prepared in accordance with the manufacturer’s protocols. Briefly, membranes were incubated with array buffer 6 for 1 h on a rocking platform shaker. The samples were prepared by adding up to 1 mL of array buffer 4 to two separate tubes and then 15 µL of reconstituted cytokine detection antibody cocktail (> 40 human cytokines) to each prepared sample and incubating for 1 h. Then, the array buffer 6 was removed and replaced with the sample/antibody mixtures and incubated at 4˚C overnight. The following day, membranes were washed thrice and then incubated with streptavidin-HRP (1:2000) for a period of 30 min at room temperature on a rocking platform shaker. Following extensive washes, 1 mL of prepared chemi reagent mix was placed onto each membrane, and the positive signals were identified by placing the transparency overlay template on the array image and aligning it with pairs of reference spots in the three corners of each array. Reference spots were incorporated to demonstrate that the array is incubated with streptavidin-HRP during the assay procedure. Pixel densities (average signals of a pair of duplicates which represent each cytokine) detected with iBright were analyzed by ImageJ and the average background subtracted from each spot [16].

### Interleukin-6 (IL-6) ELISA kit

A human ELISA kit (#ab178013, Abcam) was used to assess the concentration of IL-6 released in the media. Specifically, samples and standard curve were added to the plate with the capture and detector antibodies, allowing a sandwich complex to form in a single step. Following a 60-minute incubation period, the plate was washed. Finally, the TMB development solution was incorporated, followed by the stop solution. Plates were measured at 450 nm using a Spark microplate reader, and data are reported as pg/mL interpolated with the standard curve.

### Monocyte chemoattractant protein-1 (MCP-1) ELISA kit

A human ELISA kit (#BMS281, ThermoScientific) was used to assess the concentration of MCP-1 cytokine released in the media. The protocol for the human MCP-1 ELISA kit involves several key steps. First, the microplate strips were washed, and standard dilutions were prepared. Samples and reagents were then incubated for 2 h at room temperature. After incubation, the strips were washed again, and a substrate solution was added. This is followed by a 10-minute incubation period to develop color reactions. Finally, the absorbance was read at 450 nm using a Spark microplate reader, and data are reported as ng/mL interpolated with the standard curve.

### Macrophage inhibitory cytokine-1 (MIC-1) ELISA kit

A human ELISA kit (#EHGDF15, ThermoScientific) was used to assess the concentration of MIC-1 cytokine released in the media. First, the microplate strips were washed, and standard dilutions were prepared. Subsequently, samples and reagents were incubated for 2 h at room temperature. After incubation, the strips were washed once more, and a substrate solution was added. Subsequently, a 10-minute incubation period is required to develop the color reaction. Finally, the absorbance was measured at 450 nm using a Spark microplate reader, and data are reported as pg/mL interpolated with the standard curve.

### Active nuclear factor kappa B (p-NFkB) ELISA kit

NFkB p65 (pS536) ELISA kit (#ab176647, Abcam) was used to investigate the levels of the active form (phosphorylated) of NFkB in HUVEC upon different conditions. Specifically, the plate was designed to contain the entire complex capture (antibody/analyte/detector antibody), which had been immobilized via immunoaffinity of an anti-tag antibody coating the well. Samples were added to the wells, followed by the antibody mix. After extensive washes, TMB substrate was added, followed by the stop solution, and the intensity was read at 450 nm using a Spark microplate reader.

### Statistical analysis

**Fig. 1:**
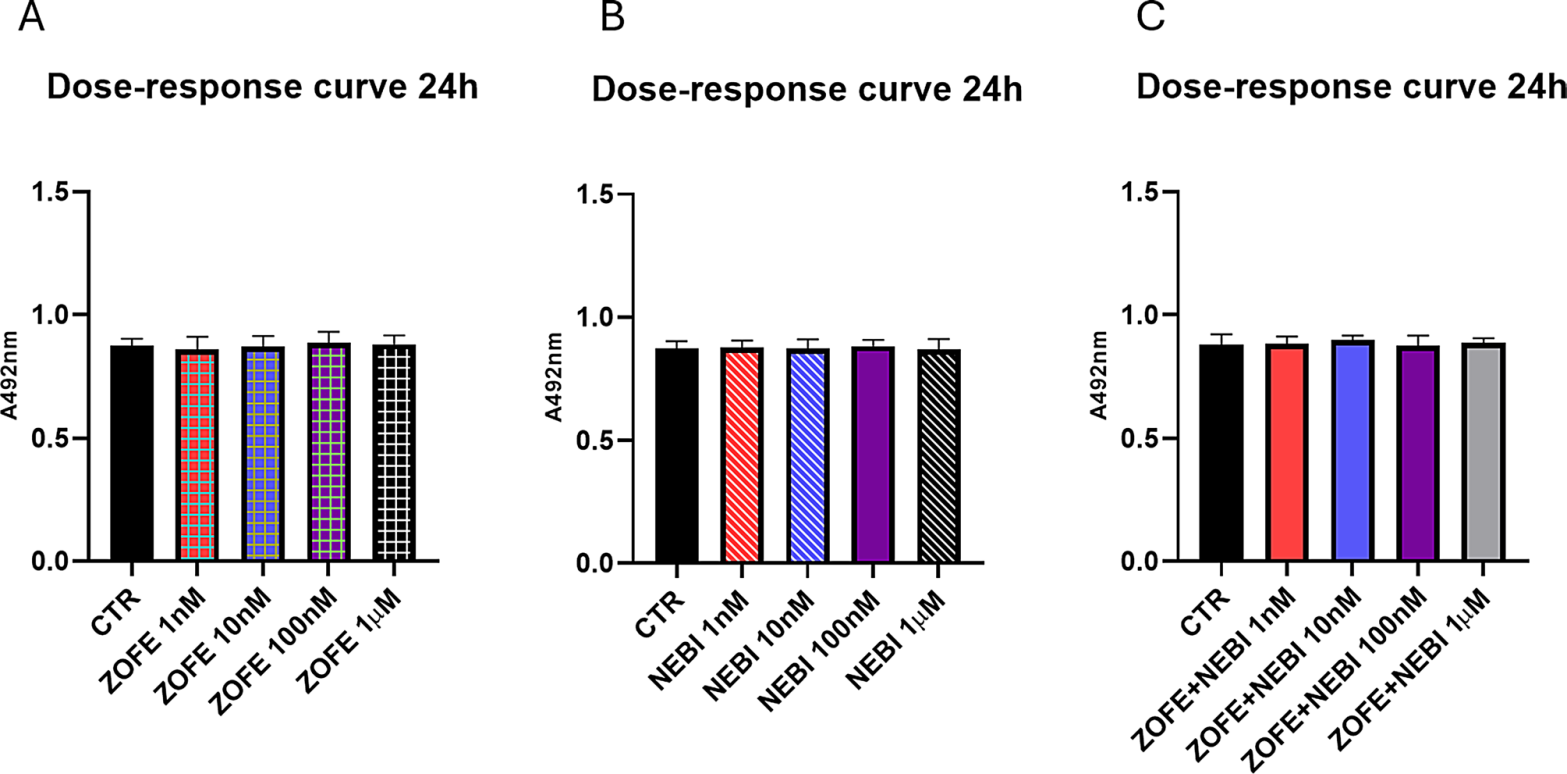
Dose-response curves. Viability assay of human umbilical vein endothelial cells (HUVEC) cells treated with **A**) zofenoprilat (ZOFE) or **B**) nebivolol (NEBI), **C**) combination for 24 h at a concentration ranging 1nM-1µM. Viability was assessed using the cell titer one solution cell proliferation assay. Data are expressed as mean ± SD of three independent experiments (*N* = 3). Statistical analysis: one-way ANOVA with Tukey’s post hoc test: ns, not significant. Abbreviations: TNFα, tumor necrosis factor-α; HUVEC, human umbilical vein endothelial cell; ZOFE, zofenoprilat; NEBI nebivolol; CTR, untreated control

**Fig. 2:**
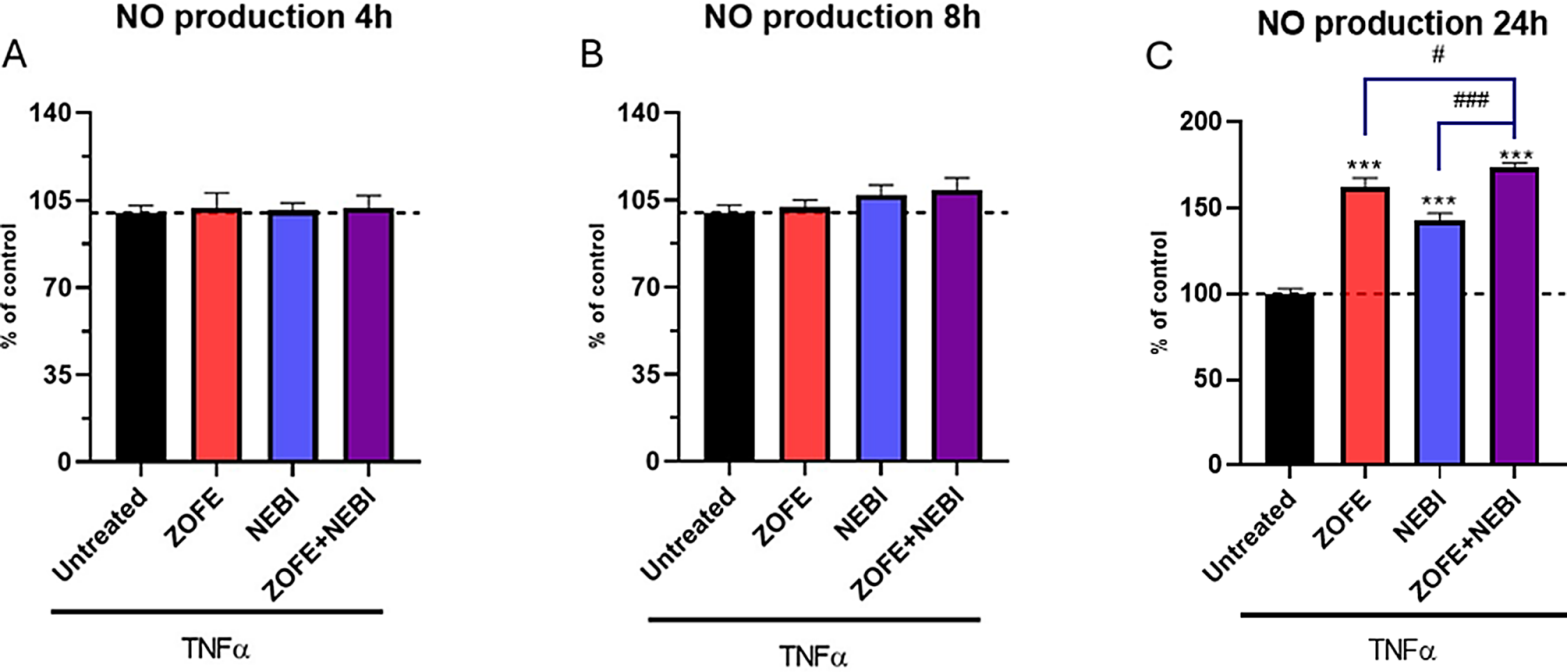
Nitric oxide (NO) analysis. Levels of NO were measured in HUVEC cell lysates after stimulation with tumor necrosis factor-α (TNFα 10 ng/ml) and different treatments with ZOFE (10⁻⁸ M) and NEBI (10⁻⁸ M), at 4 h, 8 h, and 24 h. Data are mean ± SD of three independent experiments (*N* = 3). Statistical analysis: two-way ANOVA with Tukey’s post hoc test; ***p < 0.0001, ** *p* < 0.005 vs. CTR; #<0.05 vs. ZOFE + NEBI. The dotted line represents 100% of control. Abbreviations: HUVEC, human umbilical vein endothelial cell; ZOFE, zofenoprilat; NEBI nebivolol; NO, nitric oxide; TNFα, tumor necrosis factor-α; CTR, untreated control

**Fig. 3:**
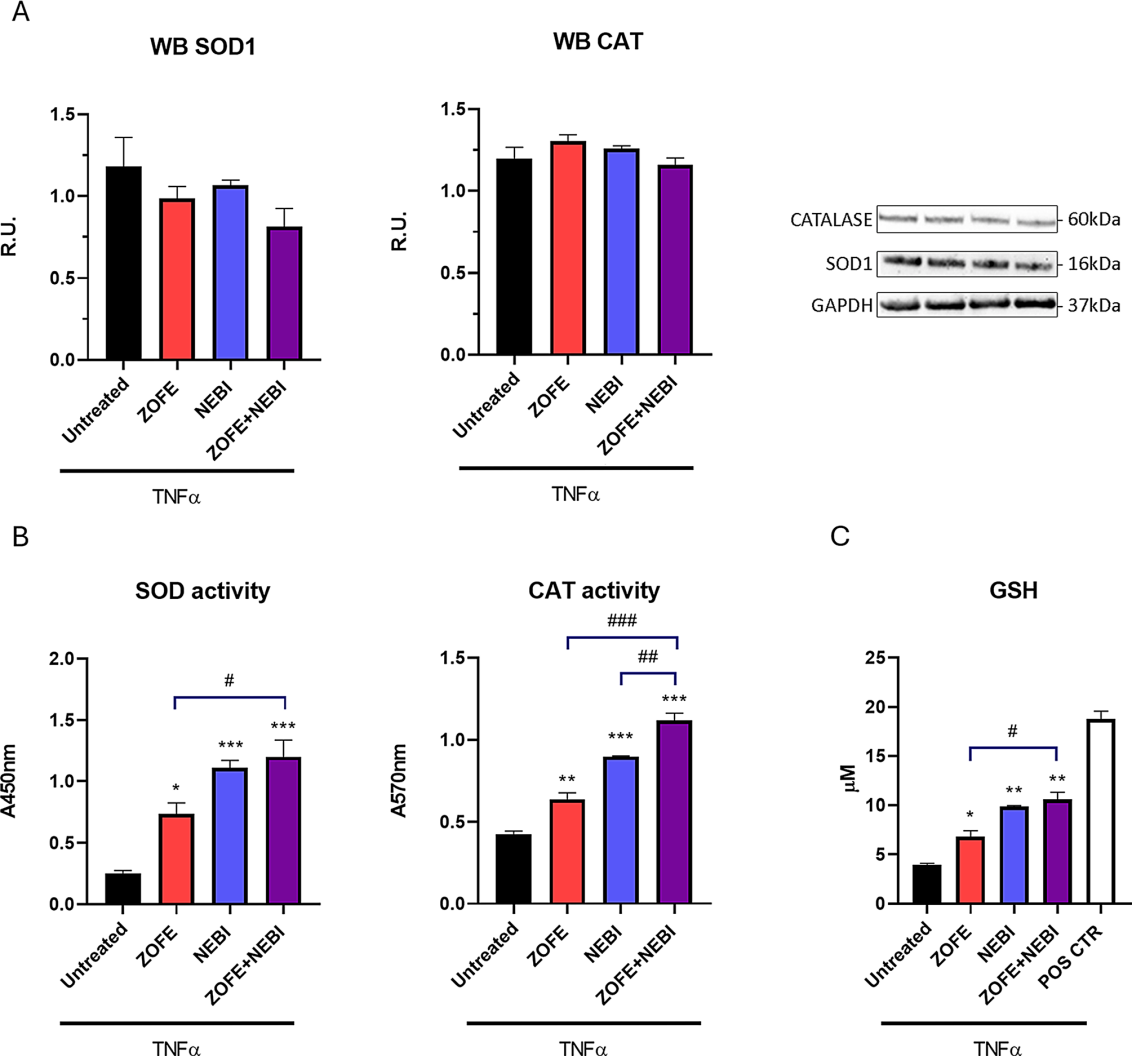
Oxidative stress analysis in TNFα-stimulated HUVECs. **A**) Western blot and relative representative figures for CAT and SOD protein levels in HUVEC upon different conditions for 24 h. **B**) SOD and CAT enzyme activities of TNFα-HUVEC upon different conditions. **C**) GSH assay in TNFα-stimulated HUVEC upon different conditions for 24 h. Data are mean ± SD of three independent experiments (*N* = 3). Statistical analysis: two-way ANOVA with Tukey’s post hoc test; *p < 0.05, **p < 0.005 ***p < 0.0005 vs. CTR; #*p* < 0.05, ##*p* < 0.005 ###*p* < 0.0005 vs. ZOFE + NEBI. Abbreviations: HUVEC, human umbilical vein endothelial cell; ZOFE, zofenoprilat; NEBI nebivolol; CAT, catalase; SOD, superoxide dismutase; GSH, glutathione; TNFα, tumor necrosis factor-α; CTR, untreated control

**Fig. 4:**
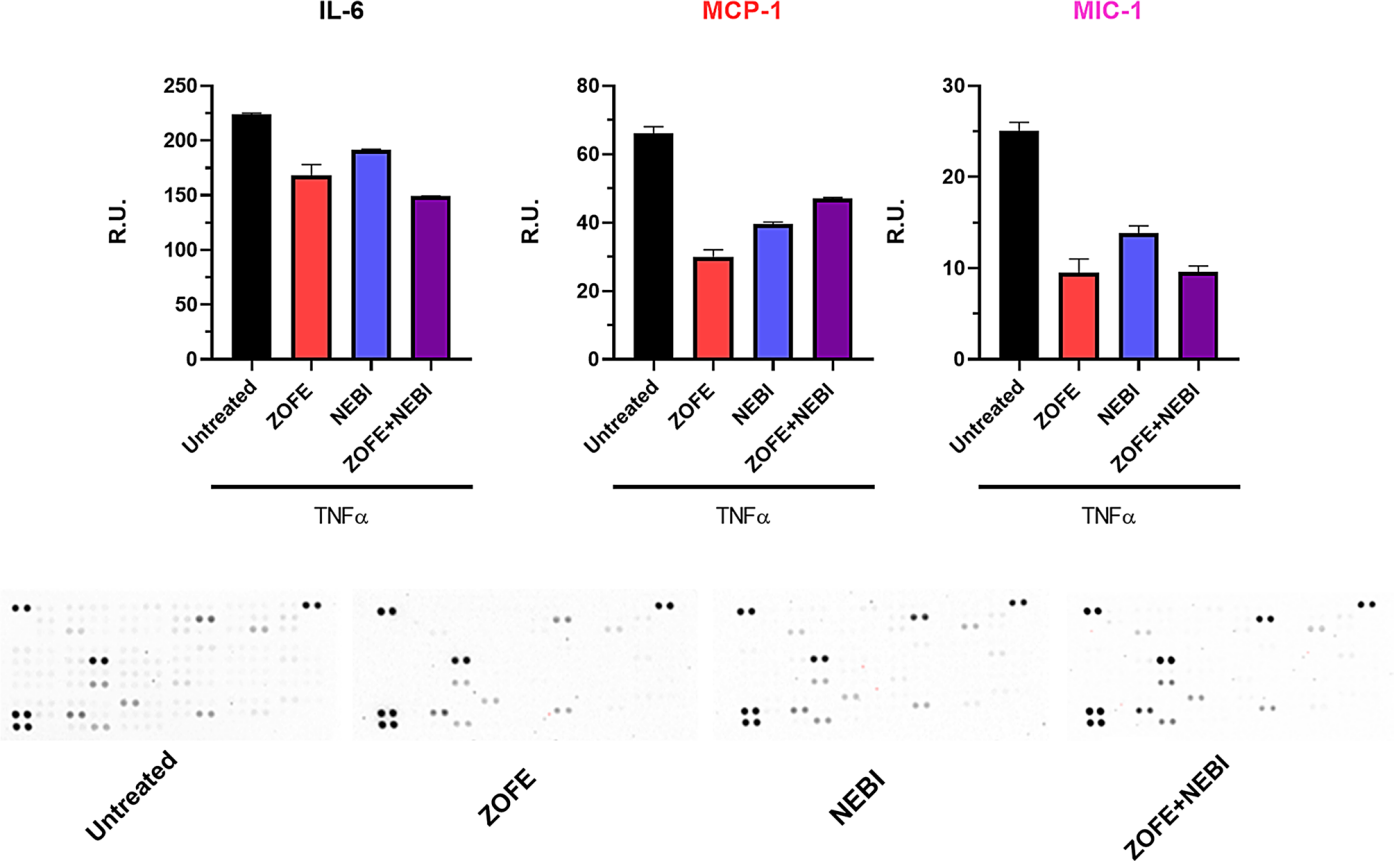
Inflammatory status analysis in TNFα-stimulated HUVECs. Proteome profiler human XL cytokine array of HUVEC lysates treated with ZOFE (10⁻⁸ M), NEBI (10⁻⁸ M), or their combination for 24 h in the presence of TNFα (10 ng/mL) (*Statistical analysis for this assay is not usually performed*). Abbreviations: TNFα, tumor necrosis factor-α; HUVEC, human umbilical vein endothelial cell; CTR, untreated control; ZOFE, zofenoprilat; NEBI, nebivolol

**Fig. 5:**
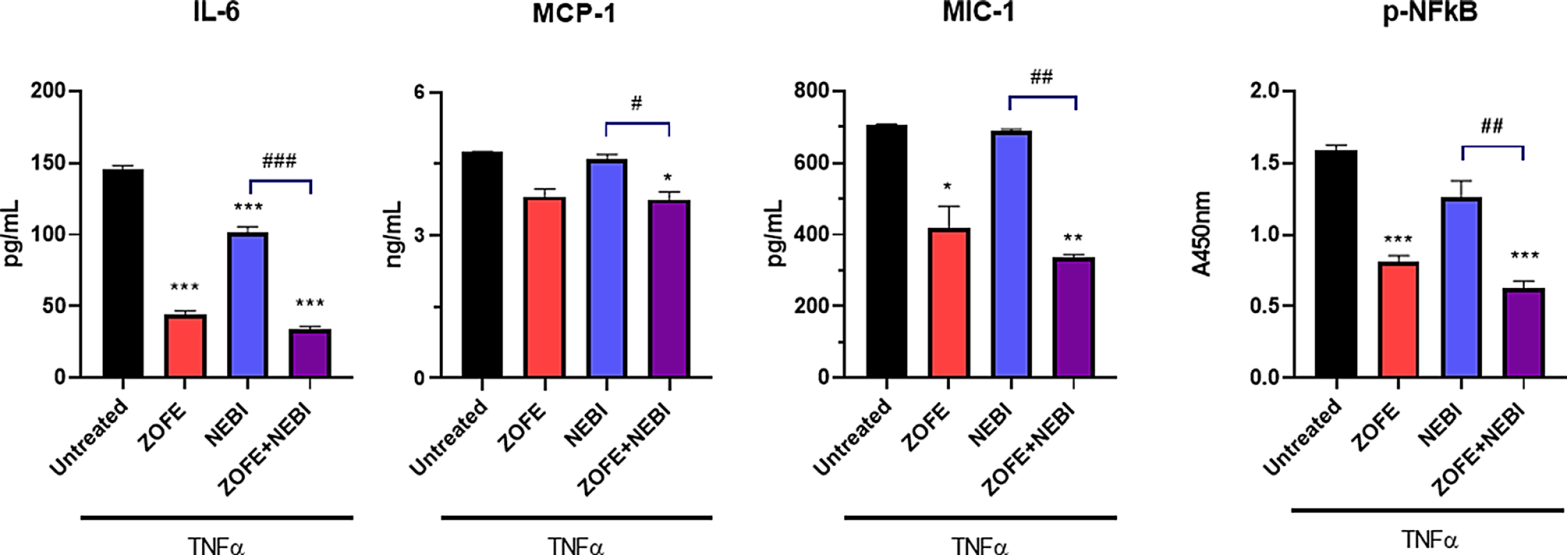
ELISA assay for the pro-inflammatory cytokines IL-6, MCP-1, and MIC-1, and the active form of NFkB. ELISA quantification of IL-6, MCP-1, MIC-1, and phosphorylated NFκB (p-NFκB) in HUVECs treated with ZOFE (10⁻⁸ M), NEBI (10⁻⁸ M), or their combination for 24 h in the presence of TNFα (10 ng/mL). Data are expressed as mean ± SD of three independent experiments (*N* = 3). Statistical analysis: two-way ANOVA with Tukey’s post hoc test; *p < 0.05, **p < 0.005, ***p < 0.0005 vs. CTR; #*p* < 0.05, ##*p* < 0.005, ###*p* < 0.0005 vs. ZOFE + NEBI

Data are mean ± SD of 3 different experiments (*N* = 3). All experiments were performed using three independent biological replicates (*n* = 3), each derived from separate cell culture preparations. For each biological replicate, technical duplicates were included to ensure reproducibility and consistency across assays. Statistical analyses were conducted using one-way ANOVA for data set reported in Fig. 1. Two-way ANOVA was performed to evaluate the main effects of ZOFE and NEBI and their interaction, followed by Tukey’s post hoc test when the interaction was statistically significant (Figs. 2, 3, 4 and 5). Analyses were performed with GraphPad Prism software, and statistical significance was set at *p* < 0.05.

## Results

### Effects of ZOFE and NEBI on cell viability

In the first set of experiments, a dose-response curve (1nM-1µM) of human umbilical vein endothelial cells (HUVEC) treated with ZOFE and nebivolol NEBI for 24 h was performed (Fig. 1A-B). Neither drug nor the concentrations tested affected cell viability (ZOFE 24 h F_4,10_=0.18, *p* = 0.94; NEBI 24 h F_4,10_=0.07, *p* = 0.99). All compounds were used at a concentration of 10^− 8^ M (10nM), as previously reported by *Desideri et al.*,* 2008*. We then performed a cell viability assay for the combined treatment of ZOFE and NEBI (F_4,10_=0.23, *p* = 0.91), and, in this case, there were no significant changes as well (Fig. 1C).

To model endothelial dysfunction, HUVEC cells were subjected to TNFα at 10 ng/ml for 24 h and then treated with the drugs alone or in combination.

### Nitric oxide (NO) production in TNFα-challenged HUVEC upon different conditions

NO production in HUVEC cells is a crucial process for vascular function and regulation. Additionally, NO possesses anti-inflammatory and anti-thrombotic characteristics, which are beneficial for overall cardiovascular well-being [17, 18].

NO production in HUVEC cells is a crucial process for vascular function and regulation, and NO also exerts anti-inflammatory and anti-thrombotic effects. NO production was analyzed in TNFα-challenged HUVECs treated with ZOFE, NEBI, and their combination by measuring NO metabolites in cell lysates at 4 h, 8 h, and 24 h (Fig. 2). At 4 h, a two-way ANOVA revealed no significant effect of ZOFE (F(1,8) = 0.574, *p* = 0.471), no significant effect of NEBI (F(1,8) = 1.796, *p* = 0.217), and no significant ZOFE×NEBI interaction (F(1,8) = 2.121, *p* = 0.183). At 8 h, a two-way ANOVA revealed a significant effect of ZOFE (F(1,8) = 8.588, *p* = 0.0190), a significant effect of NEBI (F(1,8) = 16.389, *p* = 0.00369), and no significant interaction (F(1,8) = 0.857, *p* = 0.382). Post hoc comparisons (Tukey) indicated a significant increase versus untreated only for the combined treatment (ZOFE + NEBI; 114.0% of untreated; adjusted *p* = 0.0050). At 24 h, a two-way ANOVA revealed a significant effect of ZOFE (F(1,8) = 885.76, *p* = 1.76 × 10⁻9), a significant effect of NEBI (F(1,8) = 233.65, *p* = 3.33 × 10⁻7), and a significant ZOFE×NEBI interaction (F(1,8) = 69.10, *p* = 3.31 × 10⁻5). Post hoc comparisons (Tukey) showed that ZOFE (163.4% of untreated), NEBI (139.3% of untreated), and ZOFE + NEBI (175.0% of untreated; highest increase) significantly increased NO production versus untreated (adjusted *p* < 0.0001 for each comparison). Based on these results, 24 h was selected as the time point for subsequent experiments.

### Oxidative stress analyses in TNFα-challenged HUVEC upon different conditions

We then focused on the oxidative stress response of TNFα-challenged HUVECs under different conditions. The first line of defense, antioxidant enzymes, superoxide dismutase (SOD) and catalase (CAT), are responsible for counteracting oxidative stress by detoxifying reactive oxygen species. Cu/Zn superoxide dismutase (SOD1) transforms superoxide radicals into molecular oxygen and hydrogen peroxide through redox reactions, while CAT neutralizes hydrogen peroxide by converting it into water and O₂.

In our experimental conditions, two-way ANOVA showed that SOD1 protein levels were not significantly modulated by ZOFE (F(1,4) = 3.92, *p* = 0.12), NEBI (F(1,4) = 1.62, *p* = 0.27), nor by their interaction (F(1,4) = 0.06, *p* = 0.81) (Figs. 3A and S1). Likewise, CAT protein levels were not significantly modulated by ZOFE (F(1,4) = 0.0090, *p* = 0.93), NEBI (F(1,4) = 0.87, *p* = 0.40), nor by their interaction (F(1,4) = 5.180, *p* = 0.0852) (Figs. 3A and S1). Therefore, we assessed enzyme activities (Fig. 3B). For SOD activity, a two-way ANOVA revealed a significant effect of ZOFE (F(1,8) = 15.75, *p* = 0.0041), a significant effect of NEBI (F(1,8) = 92.07, *p* = 0.000012), and a significant ZOFE×NEBI interaction (F(1,8) = 12.62, *p* = 0.0075), and post hoc comparisons showed significant differences versus untreated for ZOFE (adjusted *p* = 0.0032), NEBI (adjusted *p* = 0.0001), and ZOFE + NEBI (adjusted *p* = 0.0001). For CAT activity, a two-way ANOVA revealed a significant effect of ZOFE (F(1,8) = 47.93, *p* = 0.00012) and NEBI (F(1,8) = 233.68, *p* = 3.33 × 10⁻7), with no interaction (F(1,8) = 0.018, *p* = 0.89), and post hoc comparisons showed significant differences versus untreated for ZOFE (adjusted *p* = 0.0059), NEBI (adjusted *p* < 0.0001), and the combined treatment ZOFE + NEBI (adjusted *p* < 0.0001), with NEBI alone and the combined treatment showing the strongest effects, suggesting a stronger antioxidant activity and a higher protection of endothelial functions.

In HUVECs, adequate levels of GSH are essential for preserving endothelial function and preventing apoptosis induced by oxidative stress [19]. Thus, we decided to assay GSH in our endothelial dysfunction model upon ZOFE and/or NEBI treatment (Fig. 3C).

A two-way ANOVA revealed a significant effect of ZOFE (F(1,8) = 13.03, *p* = 0.0069), a significant effect of NEBI (F(1,8) = 96.57, *p* = 0.000010), and a non-significant ZOFE×NEBI interaction (F(1,8) = 4.42, *p* = 0.069). Post hoc comparisons (Tukey) revealed that ZOFE, NEBI, and ZOFE + NEBI significantly increased GSH versus untreated (adjusted *p* = 0.016, *p* = 0.0001, and *p* = 0.0001, respectively), with no difference between NEBI and ZOFE + NEBI.

### Inflammatory pathway analysis of HUVEC upon different conditions

We then focused on the inflammatory pathway, starting with the proteome profiler assay, which allows for the screening of many cytokines, chemokines, growth factors, and other soluble proteins in cell culture supernatants. It is notable that in TNFα-HUVEC (untreated), there are multiple spots compared to other conditions. Interestingly, ZOFE was able to reduce the levels of inflammatory mediators, such as IL-6 (1), MCP-1 (2), and MIC-1 (3) (Fig. 4) more effectively compared to NEBI.

To strengthen these results, we performed ELISA assays for IL-6 (a key cytokine involved in vascular disorders and endothelial dysfunction), MCP-1, and MIC-1 (Fig. 5) under the same experimental conditions. In parallel, another crucial protein, NFkB (active form, phosphorylated-NFkB), was analyzed (Fig. 5). For IL-6, a two-way ANOVA revealed a significant effect of ZOFE (F(1,8) = 757.68, *p* = 3.27 × 10⁻9), a significant effect of NEBI (F(1,8) = 79.57, *p* = 1.98 × 10⁻5), and a significant ZOFE×NEBI interaction (F(1,8) = 29.48, *p* = 0.0006); post hoc comparisons (Tukey) showed that ZOFE, NEBI, and combined treatment significantly reduced IL-6 versus untreated (adjusted *p* < 0.0001 for all), while ZOFE + NEBI did not differ from ZOFE alone (adjusted *p* = 0.14). For MCP-1, a two-way ANOVA revealed a significant effect of ZOFE (F(1,8) = 109.59, *p* = 0.000006), a significant effect of NEBI (F(1,8) = 14.45, *p* = 0.005), and a non-significant interaction (F(1,8) = 1.25, *p* = 0.29); post hoc comparisons showed significant reductions versus untreated for ZOFE (adjusted *p* = 0.0002), NEBI (adjusted *p* = 0.034), and ZOFE + NEBI (adjusted *p* < 0.0001). For MIC-1, a two-way ANOVA revealed a significant effect of ZOFE (F(1,4) = 107.88, *p* = 0.00049), with no significant effect of NEBI (F(1,4) = 2.59, *p* = 0.18) and no interaction (F(1,4) = 1.15, *p* = 0.35); post hoc comparisons showed significant reductions versus untreated for ZOFE (adjusted *p* = 0.0095) and the combination ZOFE + NEBI (adjusted *p* = 0.0037), but not for NEBI (adjusted *p* = 0.98). For phosphorylated NFkB, a two-way ANOVA revealed a significant effect of ZOFE (F(1,4) = 51.87, *p* = 0.0019), with no significant effect of NEBI (F(1,4) = 0.79, *p* = 0.43) and no interaction (F(1,4) = 0.16, *p* = 0.70); post hoc comparisons showed significant reductions versus untreated for ZOFE (adjusted *p* = 0.019) and ZOFE + NEBI (adjusted *p* = 0.016), but not for NEBI (adjusted *p* = 0.80).

## Discussion and conclusions

The present study investigated the combined effects of ZOFE and NEBI on endothelial function, focusing on their anti-inflammatory and antioxidant properties. Our findings suggest that the combination of these two drugs (which is well consolidated in clinical practice) [14] offers a potentially potent therapeutic strategy for managing hypertension and protecting vascular health.

ZOFE, an ACE inhibitor, demonstrated significant anti-inflammatory activities in our study. This is consistent with previous research indicating that ACE inhibitors can reduce inflammatory markers by decreasing angiotensin II levels, which are known to promote inflammation [20, 21]. The reduction in inflammatory cytokines observed with ZOFE treatment highlights its potential in mitigating vascular inflammation, a key factor in the pathogenesis of hypertension and atherosclerosis [22, 23].

The anti-inflammatory effects observed with ZOFE in this study may be explained by its established pharmacological properties. As an ACE inhibitor, ZOFE lowers angiotensin II levels, which helps reduce pro-inflammatory signaling. Its sulfhydryl group may also enhance bradykinin-mediated vasodilation and promote nitric oxide release, both of which are associated with anti-inflammatory responses. In addition, ZOFE has been reported to influence NFκB activity, a central regulator of cytokine production and endothelial activation. Our findings confirm this mechanism, demonstrating its involvement in the context of TNFα-induced endothelial inflammation. Although further studies are needed to fully elucidate these pathways, the results support the anti-inflammatory potential of ZOFE through multiple interconnected mechanisms.

On the other hand, NEBI, a third-generation beta-blocker, exhibited strong antioxidant effects, primarily through the enhancement of NO, a crucial molecule for maintaining endothelial function and reducing oxidative stress. Such properties of nebivolol make it a preferred option when a beta-blocker is chosen according to current European Guidelines for the management of hypertension [10, 11]. Our results show that NEBI significantly counteracted oxidative stress, modulating the main antioxidant defenses (SOD, CAT, and GSH). Cells protect themselves against oxidative stress primarily through the upregulation of various antioxidant genes. Among intracellular antioxidant molecules, reduced glutathione (GSH) is the most abundant non-protein thiol. GSH helps maintain a reduced cellular environment, facilitating the removal of potentially toxic electrophiles and metals, and thereby safeguarding cells from harmful oxygen products. Oxidative stress is a major contributor to endothelial dysfunction and cardiovascular disorders; thus, our results strengthen existing published literature that highlights the unique properties of nebivolol in improving endothelial health [24, 25].

Although NEBl is widely recognized for its antioxidant properties associated with enhanced NO production, our findings indicate that this mechanism alone may not fully explain its biological effects. Interestingly, ZOFE elicited even higher NO levels at the 24-hour time point; however, it did not enhance antioxidant enzymes such as SOD, CAT, or GSH to the same extent. This observation implies that NEBI may engage additional pathways beyond NO signaling. One plausible mechanism involves β3-adrenergic receptor activation, which has been associated with cellular antioxidant responses. Moreover, NEBI might influence the transcriptional regulation of antioxidant defenses through factors like Nrf2, contributing to the upregulation of protective enzymes. It is also possible that the relationship between NO availability and antioxidant enzyme induction is not linear, but rather influenced by cellular context, timing, or upstream signaling dynamics. These considerations highlight the multifactorial nature of NEBI’s antioxidant action and warrant further investigation.

In this regard, a limitation of this study is the lack of direct assessment of oxidative damage. While we measured antioxidant defenses, we did not include markers such as ROS, MDA, or DNA/protein oxidation. Future studies should address this to better define redox balance.

The most interesting outcome of our research is that the combination of ZOFE and NEBI resulted in a potentiated effect, enhancing both anti-inflammatory and antioxidant activities. This dual mechanism of action provides a comprehensive approach to protecting endothelial cells and improving vascular function. These results suggest that the combined therapy not only lowers blood pressure more effectively and is safe (completed trial: NCT05257148 Masaccio trial) but may also offer greater protection against endothelial damage compared to monotherapy with either drug alone.

The additive/synergistic effects observed in this study suggest that the combination of ZOFE and NEBI could be particularly beneficial for patients with hypertension, especially those with coexisting inflammatory and oxidative stress-related conditions. It is worth emphasizing that IL-6 has a predominant role in the pathophysiology of cardiovascular alterations. In fact, angiotensin II is known to act on several cytokines, including IL-6, the activation of which is associated with increased arterial stiffness [26]. In addition, more and more evidence underlines the contribution of MIC-1 to adverse clinical events related to hypertension and cardiovascular disease [27]. Considering the above, the data obtained on the anti-inflammatory effect following treatment with both compounds takes on even more relevance. This combination therapy could be potentially beneficial in patients with cardiovascular diseases by addressing multiple pathogenic pathways simultaneously. We used HUVECs as a standard endothelial model due to their accessibility and reproducibility. However, we acknowledge that arterial endothelial cells, such as human aortic endothelial cells HAECs, would better reflect the vascular environment where ZOFE and NEBI exert their therapeutic effects. This limitation has been noted, and future studies will include arterial models to enhance translational relevance.

Obviously, in vitro models present several limitations and cannot fully replicate the complexity of in vivo systems. Nevertheless, in vitro models remain a valuable tool for studying the therapeutic potential of compounds [28].

Further clinical and preclinical investigations are required to confirm and validate the findings observed in this study.

While our study provides valuable insights into the combined effects of ZOFE and NEBI, which add on top of the consolidated use in common clinical practice [14], further research is needed to confirm these findings in clinical settings.

In conclusion, the combination of ZOFE and NEBI offers a potentially promising therapeutic approach for managing hypertension and protecting vascular health. By leveraging their complementary anti-inflammatory and antioxidant properties, this combination therapy may significantly improve clinical outcomes for patients with cardiovascular diseases.

## Supplementary Information

Below is the link to the electronic supplementary material.

Supplementary Material 1: **Figure S1**. Full Western blot images corresponding to Figure 3A: A) Catalase, B) SOD1, and C) GAPDH in HUVECs upon different experimental conditions for 24 hours.Abbreviations: TNFα, tumor necrosis factor-α; HUVEC, human umbilical vein endothelial cell; ZOFE, zofenoprilat; NEBI nebivolol; IL-6, interleukin-6; MCP-1, monocyte chemoattractant protein-1; MIC-1, macrophage inhibitory cytokine-1; p-NFκB, active form of nuclear factor kappa B; NFκB, nuclear factor kappa B; TNFα, tumor necrosis factor-α; CTR, untreated control.

## Data Availability

The data that support the findings of this study are available on request from the corresponding authors.
