# Supplementary figures and images for: Potentiating anti-inflammatory and antioxidant effects in vitro: the combined action of zofenoprilat and nebivolol
Source: Pharmacol Rep. 2026 Feb 3;78(2):546–57. doi: 10.1007/s43440-026-00833-x (PMC12975774; doi:10.1007/s43440-026-00833-x)

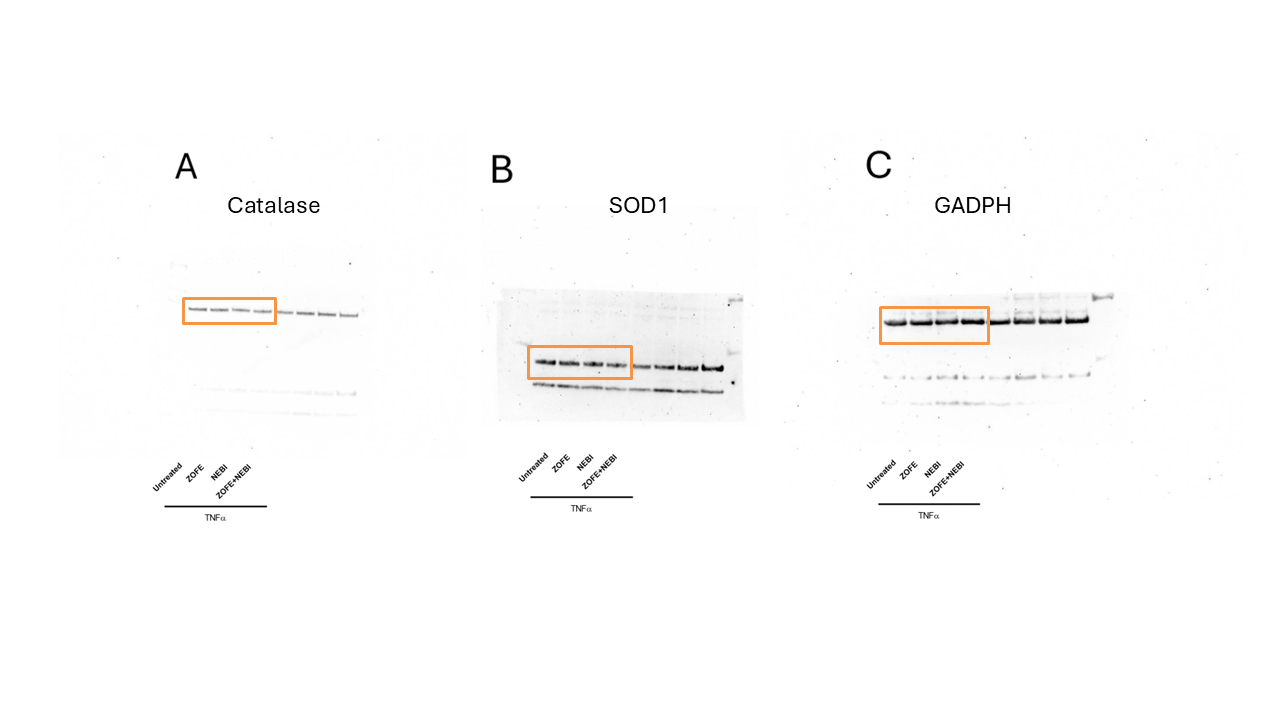

Supplement: Supplementary file 1 — Supplementary Material 1: Figure S1. Full Western blot images corresponding to Figure 3A: A) Catalase, B) SOD1, and C) GAPDH in HUVECs upon different experimental conditions for 24 hours.Abbreviations: TNFα, tumor necrosis factor-α; HUVEC, human umbilical vein endothelial cell; ZOFE, zofenoprilat; NEBI nebivolol; IL-6, interleukin-6; MCP-1, monocyte chemoattractant protein-1; MIC-1, macrophage inhibitory cytokine-1; p-NFκB, active form of nuclear factor kappa B; NFκB, nuclear factor kappa B; TNFα, tumor necrosis factor-α; CTR, untreated control. [file 43440_2026_833_MOESM1_ESM.tif]
